# *Stenotrophomonas maltophilia* complex: A broadly distributed emerging pathogen

**DOI:** 10.1371/journal.ppat.1013422

**Published:** 2025-08-21

**Authors:** Cristian V. Crisan, Joanna B. Goldberg

**Affiliations:** 1 Department of Pediatrics, Division of Pulmonary, Asthma, Cystic Fibrosis, and Sleep, Emory University School of Medicine, Atlanta, GeorgiaUnited States of America; 2 Emory+Children’s Center for Cystic Fibrosis and Airway Disease Research, Emory University School of Medicine, Atlanta, GeorgiaUnited States of America; Tufts Univ School of Medicine, UNITED STATES OF AMERICA

## What is *Stenotrophomonas maltophilia* complex?

*Stenotrophomonas maltophilia* complex (Smc) is a group of globally distributed gram-negative Gammaproteobacteria from the *Lysobacterales* order that colonize soil, water, and plant ecosystems [1]. In addition to these natural environments, Smc strains are found in anthropogenic settings like hospitals, especially in intensive care units, and other healthcare facilities [1,2]. Isolates exhibit a high level of genomic diversity and have been classified into 23 monophyletic lineages [1]. Sm6, also known as *S. maltophilia sensu stricto*, is the most common lineage [1]. K279a (isolated from the blood of a cancer patient) belongs to the Sm6 lineage and is one of the best characterized Smc strains [3]. Lineages that are more closely related to Sm6 (Sm1-Sm5 and Sm7-Sm18) are classified as *S. maltophilia sensu lato*, while Sgn1–4 (*Stenotrophomonas* genospecies 1–4) are the most distantly related to Sm6 [1]. Multiple lineages, including Sm2, Sm4a, Sm4b, Sm6, and Sm13 are associated with human infections, while Sgn1–3 are primarily recovered from environmental sources [1].

## Why is Smc clinically important?

**Fig 1:**
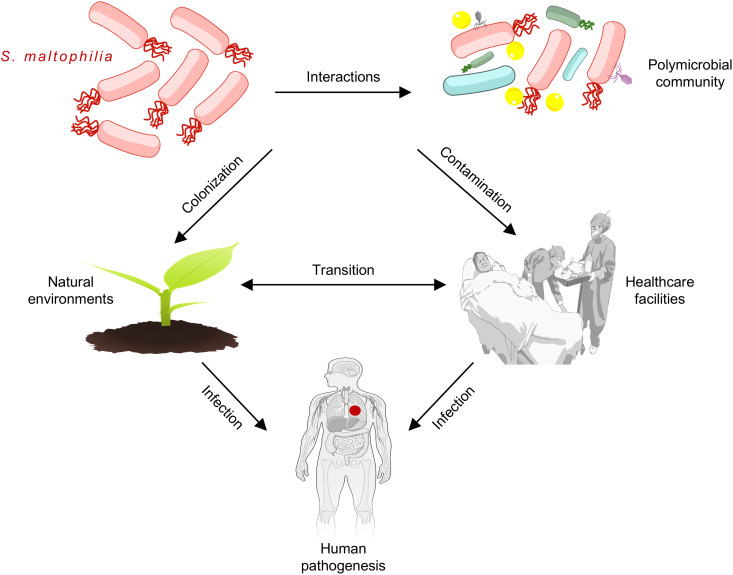
Smc isolates are versatile and adaptable. The ability of Smc bacteria to survive in polymicrobial communities, to colonize natural environments like soil and water, and to contaminate surfaces in healthcare settings may contribute to the potential to infect susceptible people and cause disease. Images used to build this figure are freely available and can be modified or adapted under the National Institutes of Health NIH BIOART Source Public Domain License (https://bioart.niaid.nih.gov/bioart/42, https://bioart.niaid.nih.gov/bioart/206, and https://bioart.niaid.nih.gov/bioart/519), Open Clipart Public Domain License, (https://openclipart.org/detail/62785/virus), and Pixabay Content License (https://pixabay.com/vectors/ground-nature-plant-spring-2022491/).

Many Smc isolates are opportunistic pathogens that can infect the lungs, brain, skin, urinary tract, eyes, and blood [4–8]. Due to this bacterium’s ability to contaminate and colonize medical equipment and other healthcare surfaces, nosocomial infections are a significant concern (Fig 1) [9]. Blood infections are especially dangerous and can have mortality rates above 65%; in a study with over 1600 bacteremia patients, Smc was the most frequently detected carbapenem-resistant gram-negative pathogen [5,10]. Bacteria from this group can cause serious health problems for people with cystic fibrosis (CF), chronic obstructive pulmonary disease (COPD), COVID-19, or cancer [4,9,11,12]. CF and COPD patients that acquire Smc infections are more likely to suffer from worse health outcomes, have higher mortality, and lower pulmonary functions [11,12]. Smc isolates had the highest rates of multidrug resistance among bacterial pathogens that infect COVID-19 patients [4]. Multiple risk factors, including mechanical ventilation, venous catheters, tracheotomies, and previous exposure to antibiotics can contribute to Smc infections [2,13,14]. Because people with weakened immune systems or other underlying conditions are the most vulnerable, some debate exists whether this bacterium is a true pathogen or if its detection represents a marker for severe disease.

Importantly, the number of Smc infections has been increasing in the last decades [15,16]. This phenomenon could be explained by a higher number of admissions to critical care units and increasing rates of underlying conditions like cancer [17,18]. Furthermore, improved detection procedures that classify clinical isolates with better accuracy and a greater recognition about the pathogenic potential of Smc among clinicians could also be responsible for the reported increased infection rates [19,20].

**Fig 2:**
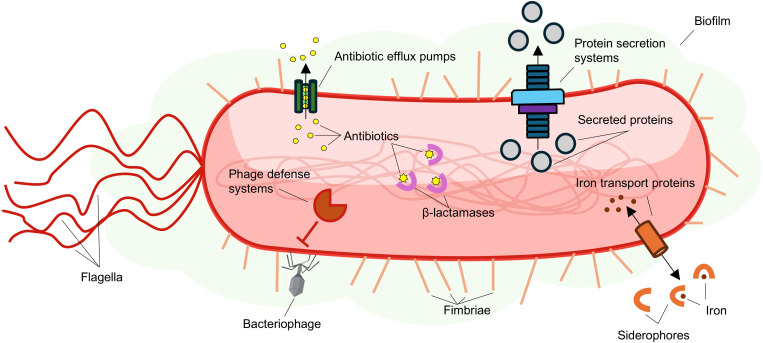
Smc strains encode proteins that contribute to virulence, survival in external environments, and resistance to antibiotics. Secreted biofilm products facilitate adherence to abiotic surfaces and eukaryotic cells, while membrane appendages like flagella and pili contribute to motility and adherence. Protein secretion systems export virulence factors and antibacterial proteins. Smc antibiotic efflux pumps and β-lactamases confer resistance to antibiotics, while putative defense systems may prevent phage infections. Siderophores and transport proteins allow iron to be transported into cells. The image used to build this figure is freely available and can be modified or adapted under the National Institutes of Health NIH BIOART Source Public Domain license (https://bioart.niaid.nih.gov/bioart/42).

Multiple factors contribute to this bacterium’s ability to adhere to surfaces (Fig 2). Biofilms are dense microbial communities that consist of cells encased in a complex matrix composed of polysaccharides, DNA, lipids, and/or proteins [21]. This matrix enhances bacterial adhesion to abiotic surfaces and eukaryotic cells. Most Smc isolates can form biofilms, but the amount of secreted extracellular matrix is variable and strain-dependent [21]. Polar flagella, which are tail-like macromolecular structures involved in motility and adhesion, are present on the surface of Smc cells and modulate host immune responses [22]. Hair-like projections distributed on the membrane (called fimbriae) also enhance the ability of this pathogen to adhere to surfaces [23].

Secreted proteins play important roles in Smc virulence. The Type II Secretion System (T2SS) is used by many pathogens to excrete proteinaceous virulence factors [24,25]. This macromolecular apparatus spans the inner membrane, periplasm, and outer membrane of gram-negative bacteria and translocates bacterial toxins to the extracellular milieu in an ATP-dependent manner [24,25]. Strain K279a exports multiple T2SS proteases that induce host cellular damage, degrade eukaryotic proteins, and modulate immune system responses [24,25]. StmPr1 is a T2SS serine protease that degrades cellular adherens and tight junctions, contributes to cell death, and induces interleukin-8 (IL-8) secretion through the protease-activated receptor 2 [25,26]. StmPr1 is the main T2SS virulence factor in K279a and is encoded by the majority of Smc strains from all lineages [1,25]. StmPr2 and StmPr3 are additional T2SS proteases secreted by K279a that degrade eukaryotic proteins and induce cellular toxicity [25,26]. StmPr1, StmPr2, and StmPr3 share sequence homology to proteases encoded by bacteria from the *Xanthomonas* genus [25,26].

K279a also encodes a functional Type IV Secretion System (T4SS) that is structurally and functionally distinct from the T2SS [27]. The T4SS assembles a large proteinaceous nanomachine that consists of an inner membrane complex connected to an outer membrane complex through a periplasmic stalk-like structure [27]. Bacterial processes like conjugation, natural transformation, and virulence factor export are mediated by the T4SS. In K279a, the T4SS apparatus induces apoptosis in macrophage cells while reducing apoptosis in human alveolar adenocarcinoma cells [27]. Although it is unclear which effector proteins are responsible for the ability of K279a to regulate eukaryotic cell death, T4SS-mediated apoptotic modulation is caspase-dependent [27].

Other secreted small molecules and protein complexes can contribute to virulence in Smc bacteria. Iron serves as a cofactor for enzymes that catalyze core metabolic reactions and is an essential nutrient that most bacterial pathogens acquire from hosts during infections [28]. Sm6 strain K279a produces siderophores and efflux pumps that maintain iron homeostasis [28]. Iron also serves as an external signal that alters expression of virulence factors, biofilm production, and oxidative stress responses [29].

Infections with bacteria from this group are difficult to treat because antibiotic resistance is widespread [4]. Multiple efflux pumps are encoded in most Smc genomes [1,30,31]. These proteinaceous complexes export fluoroquinolones, tetracyclines, trimethoprim, and sulfamethoxazole antibiotics [30–32]. Furthermore, beta-lactamases that inactivate carbapenems are also produced by Smc strains [33]. The prevalence of antibiotic resistance genes varies across Smc lineages [1]. Bacteria from all Smc lineages encode Resistance-Nodulation-Division (RND) efflux pumps, while aminoglycoside-phosphotransferases are found in approximately 60% of all sequenced genomes [1]. K279a possesses two β-lactamase genes: *blaL1* and *blaL2*; while *blaL1* is found in more than 80% of Smc genomes, *blaL2* is found in less than 65% of genomes and is absent from lineages Sm1, Sm12, Sm13, Sm16, and Sgn4 [1]. Less than 2% of all strains possess the *sul1* gene, which confers resistance to sulfonamide [1]. This variation in the prevalence of antibiotic resistance genes among Smc isolates could be explained by the high frequency of horizontal gene transfers proposed to occur among bacteria from this group [1,34].

Strain K279a produces a cell-cell communication molecule called diffusible signal factor (DSF) [35,36]. DSF positively regulates multiple phenotypes associated with pathogenicity, such as production of virulence factors, biofilm, and siderophores [35,36]. DSF also controls antibiotic resistance antibiotics by inducing β-lactamase production [35].

## How do Smc isolates interact with other microorganisms?

Smc strains have been recovered from polymicrobial infections that contain bacterial pathogens like *Pseudomonas aeruginosa*, *Staphylococcus aureus*, and *Escherichia coli* [37,38]*.* Both antagonistic and cooperative interactions have been observed between Smc and other bacteria [37–42]. Using mouse in vivo models, McDaniel and colleagues observed that *P. aeruginosa* can increase the ability of strain K279a to colonize lungs during co-cultures, suggesting that cooperative interactions exist between these two pathogens during infections [41,42]. Alio and colleagues found that K279a alters gene expression of co-cultured *P. aeruginosa* and *S. aureus* and interferes with *P. aeruginosa* quorum sensing [43]. Furthermore, clinical Smc isolates share mobile genetic elements with *P. aeruginosa*, suggesting that inter-species horizontal gene transfers occur between these two pathogens [34].

Bacteria from the Smc group also engage in antagonistic interactions, which are dependent on the identities of both attacker and target bacteria [27,38,39,44,45]. Isolates from people with CF display strong antibacterial effects against *E. coli* but are less successful at eliminating *P. aeruginosa* or *S. aureus* [38]*.* In addition to its roles in degrading eukaryotic proteins, the K279a T4SS can deliver antibacterial proteins that eliminate the plant pathogen *Xanthomonas citri*, as well as human pathogens like *E. coli* and *P. aeruginosa* [27,39,45]*.* The antibacterial activity of the K279a T4SS is likely dependent on multiple secreted protein effectors, which are neutralized by cognate immunity proteins [39,40,45]. One Smc T4SS effector degrades DNA, while others are predicted to damage the peptidoglycan cell wall or lipid membranes [39,40].

Unlike K279a, Smc strain STEN00241 (which belongs to the *sensu lato* Sm13 lineage) does not possess T4SS genes, but harbors an antibacterial Type VI Secretion System (T6SS) [44,46]. The T6SS is distinct from the T4SS and adopts a harpoon-like structure [44]. A baseplate and membrane complex span across the bacterial inner membrane, periplasm, and outer membrane to accommodate an inner tube that is decorated with a sharp tip. This inner tube is secreted and delivered along with antibacterial protein effectors into adjacent cells [44]. Similar to the T4SS, immunity proteins neutralize cognate toxins to prevent self-intoxication of bacteria that harbor T6SS effectors. STEN00241 uses the T6SS to eliminate bacterial competitors like *E. coli* and *Burkholderia cenocepacia* [44]*.* Furthermore, the T6SS also modulates interactions between STEN00241 and a co-infecting *P. aeruginosa* strain isolated from the same patient [44]. T6SS genes encoding multiple putative antibacterial effectors predicted to damage the bacterial cell envelope have been identified in STEN00241 [44].

Another antibacterial weapon used by bacteria to eliminate competitors is contact-dependent inhibition (CDI). CDI systems assemble a membrane pore through which a long, stick-like protein is secreted outside the cell [47]. Once exported, this long protein binds to a receptor on the target cell and delivers an antibacterial toxin. Sibling cells are protected because they express immunity proteins. In general, bacteria can only use CDI toxins to antagonize related cells from the same species or genus. CDI genes and toxins with diverse predicted functions are ubiquitous among Sm6 genomes [47]. A CDI toxin from a CF Sm6 isolate has antibacterial properties and is predicted to degrade tRNA molecules [47]. By contrast, strains from other Smc lineages rarely possess CDI genes, suggesting that this apparatus could have conferred bacteria from the Sm6 lineage competitive advantages over other lineages [47].

Bacteria from the Smc group also engage in interactions with fungi [43, 48]. *Candida albicans* and *Aspergillus fumigatus* are opportunistic fungal pathogens that cause infections in people with weakened immune systems due to cancer or its treatment, as well as people with CF, COPD, and those on mechanical ventilation. Sm6 strain ATCC 13637 impairs *A. fumigatus* hyphal growth, reduces the number of conidia, and causes this fungus to increase its cell wall thickness [48]. Similarly, K279a hinders growth of *C. albicans* during co-cultures and alters fungal gene expression [43].

In addition to bacteria and fungi, Smc strains interact with bacteriophages (or phages), which are viruses that infect bacterial cells [49,50]. Based on their lifestyle, phages can be either lysogenic or lytic [50]. Following infection, lysogenic phages integrate their genomes onto the bacterial DNA and replicate along with their host. By contrast, lytic phages infect bacteria, reproduce to high numbers, and kill cells to release virus particles that continue the infectious process [49,50]. Lysogenic and lytic phages from diverse viral families that infect Smc strains have been described [49,50]. Furthermore, most Smc isolates harbor genes predicted to encode an extensive arsenal of antiphage defense systems, suggesting that interactions between this bacterium and phages are frequent [51].

## Conclusions and future directions

Bacteria from the Smc group are emerging, multidrug-resistant pathogens broadly distributed in natural and anthropogenic environments. These microorganisms possess an array of factors that contribute to their ability to survive in external environments and to cause multiple types of infections. Smc isolates engage in complex interactions with other bacteria, fungi, and phages. Future work is required to understand the regulation of virulence factors and antibiotic resistance genes, the roles played by other microorganisms in modulating Smc pathogenicity, and the potential of alternative options (such as phages) to treat infections resistant to multiple antibiotics.
